# Vehicle yielding probability estimation model at unsignalized midblock crosswalks in Shanghai, China

**DOI:** 10.1371/journal.pone.0213876

**Published:** 2019-03-14

**Authors:** Jairus Odawa Malenje, Jing Zhao, Peng Li, Yin Han

**Affiliations:** Department of Traffic Engineering, University of Shanghai for Science and Technology, Shanghai, P.R. China; University of British Columbia, CANADA

## Abstract

The vehicle-pedestrian encounter at midblock crosswalks in urban centers is inevitable but the challenge to urban transportation planners is in achieving a balance between traffic flow efficiency and pedestrian safety. Vehicles are expected to yield to pedestrians who have a right of way at the midblock unsignalized crosswalks but, failure to yield causes conflicts that at times are fatal. This study investigated the effect of macroscopic factors on the vehicle yielding. Six environmental factors are considered: temporal gap size, number of traffic lanes, number of waiting pedestrians, position of pedestrian (whether on street kerb or median), traffic flow direction and presence (or absence) of monitoring ePolice. Video Data on six observed variables that influenced vehicle yielding was collected from 13 uncontrolled crosswalk locations in Shanghai city in the Peoples Republic of China. A Logit model with a 95.9% accuracy was developed to describe the vehicle yielding behavior. The results showed that gap size and number of traffic lanes had the highest influence on driver yielding decision and that drivers were more likely to yield if ePolice was present. The sensitivity analysis was conducted and appropriate recommendations on improving the pedestrians crossing safety were proposed accordingly.

## Introduction

Interaction between pedestrians and motor vehicles have continued to face increased conflicts over the years in many urban centers worldwide [1–6]. These conflicts happen when pedestrians cross a road segment, sometimes in a straight line, diagonally or zig-zag (rolling gap) and in both directions at formally designated sites (controlled or uncontrolled crosswalk) or at any site deemed convenient to the pedestrian [7]. The safety of pedestrians largely depends on their own capacity to correctly discern the traffic flow situation [8] by accepting appropriate gaps and the drivers’ willingness to yield. Creating a pedestrian crosswalk is a popular measure to enforce pedestrian safety against conflicts with vehicles, though drivers often violate the requirements of using the crosswalks [9]. However, the associated costs of setting up and running the safer and more effective traffic signal control [10, 11] and the need for traffic flow efficiency [11–13] creates a constraint on the possible number of controlled crosswalks that can be viably set up on any motorway.

The yielding behavior of drivers in response to traffic situations may be influenced by traffic-related environmental factors such as road geometry, approach speed, number of pedestrians and their behavior, legal obligations [14, 15] and their own personality traits such as gender, age, disability status, attitude and temperament [16–18]. Factors related to personality and character vary widely from one person to the next and effecting sanctions or interventions is a challenge. Therefore, this study focused on macroscopic factors affecting vehicle yielding that can be controlled by transportation administrators. The data were collected from 13 uncontrolled crosswalk locations in Shanghai, China using a video camera mounted on unmanned aerial vehicle (UAV). A Logit model was developed to describe the vehicle yielding behavior. The proposed model was validated and the sensitivity analysis was conducted.

## Literature review

To achieve the desirable quality of service on urban roads requires a careful balance between all the parties sharing the roadway. Regardless of the location where pedestrians elect to cross, vehicles should yield whenever they find pedestrians crossing to avoid a potentially tragic situation [19–22]. If the pedestrians are in a larger platoon, the vehicle may have to not only yield but also stop and wait for the crossing to clear or a gap to appear [23, 24]. On marked midblock crosswalks pedestrians have a universal right of way but, many drivers do not yield [17, 25, 26] especially when the platoon size is small [23, 27]. Sometimes pedestrians’ decision regardless of their rights, may not be prudent at a point in time when an aggressive and intransigent driver is approaching while some drivers accelerate in an effort to discourage pedestrians from attempting to cross [25].

One of the reasons that has been cited when vehicles fail to yield is a driver’s inability to judge or predict pedestrians’ intentions and actions correctly. In certain cases pedestrians hurriedly enter the road and then slow down as they cross [28] while others appear to be waiting and then suddenly start crossing which is contrary to the drivers’ concept of future expectations [24]. Hirun et al. [17, 29] found that drivers failed to yield at designated crosswalks due to ignorance of the pedestrians’ right of way but in Sweden, the law did not explicitly give pedestrians right of way but depended on driver’s goodwill and judgement [30]. In Beijing, Zhuang and Wu [13] found that the low rates of yielding were caused partly by the condescending attitude of drivers towards pedestrians. However, some studies [24, 31–33] noted that pedestrian interventions such as gestures, eye contact and smiling at the drivers produced higher rates of yielding among male drivers unlike female drivers who were comparatively less-responsive.

Approach speed of a vehicle at the crosswalk is also an important contributor to yielding or lack of it. Some studies [20, 34–37] notes that a yield can only be possible if a driver can reasonably react to the arrival of a pedestrian, given the vehicle travel speed, distance from the conflict area, and maximum (comfortable) deceleration rate for the individual driver. However, higher rates of yielding were observed when approach speed was lower against pedestrians crossing in groups. Age was also a factor with Elias (2018) noting that 71% of the drivers below 24years of age were likely to get involved in fatal accidents in Israel. Female and older drivers were also found to stop more often when approach velocity was low [38]. Furthermore, rates of yielding were likely to be higher when the distance from the pedestrian to the vehicle was higher, when pedestrians were crossing in groups, while looking at the approaching driver. It was found that jaywalkers were less likely to accept driver’s yielding behaviors, resulting in a lower yield utilization rate than permissive crossings at crosswalks [39]. An emerging dangerous multiple threat phenomena is where a vehicle in one lane yields but the obscured vehicle on the next lane doesn’t [40]. The result is a potentially fatal high speed vehicle-pedestrian conflict.

Many of the studies that have been undertaken to predict the probability of a vehicle yielding have considered a matrix of factors both environmental and personality-related to study and recommend strategies of enhancing pedestrian safety at the crosswalks. In [17, 34, 35, 41] the studies examined the interface between drivers and pedestrians, pedestrians’ road crossing and driver-yielding behaviors at unsignalized mid-block crosswalks. Hirun [17] focused on age, sex, education, experience, type of vehicle, and knowledge of pedestrian’s right-of-way law, the size of traffic gaps accepted by pedestrians and the decision on whether or not to cross the street, as well as the related determinants and the results show that over 50% of the drivers did not understand pedestrian right of way. Stapleton et al. [15] examined how yielding was effected by crossing distance, presence of median island, vehicular and pedestrian volumes, travel lane of the subject vehicle, and the subject vehicle’s position in a queue and found that yielding compliance improved substantially when crosswalk markings were used. They also found that yielding was not significantly sensitive to the particular travel lane of the subject vehicle. In [34, 41] the number of lanes and vehicle approach speed were found to be inversely proportional to the probability of yielding and the results clearly showed that speed was the major influence in the changing yield rates. In most cases, logistic regression models were used to predict the yielding probability. Houten et al. [29] studied the role of right-of-way public enforcement campaigns and found that drivers yielded more as a result of the campaign.

From the foregoing, it is vital to establish the probability of yielding to pedestrians. In this study we establish a yielding probability estimation model to predict the probability of a vehicle can yield to pedestrians at the crosswalks.

## Data collection

The “Yield or Not” represents the outcome variable that is used to predict the driver yielding behavior in this study. It is a binary variable the takes one of 2 values (yes or no, 1 or 0) based on the response of the driver.

### Analysis of potential influencing factors

There are many factors that influence the yielding decisions. In this study, the following six variables of interest were identified as having influence on the vehicle yielding decisions: gap size (seconds), number of lanes, pedestrian number (persons), position of pedestrian (0-roadside, 1-middle of the street), traffic flow direction (0-one way, 1-two way), and E-Police (0-no, 1-yes). Below is a summary of the variables in the study.

- Gap size, G_s_: It is an influencing scale-variable that represents the time gap between the oncoming vehicles in a traffic stream at a particular instance in time. It is measured in seconds.
- Number of lanes, N_L_: This is a discrete number variable that represents the number of lanes on the road segment. The number of lanes has a big effect on whether pedestrians will accept gaps or not because it has a bearing on how long one can take to traverse the road segment from the kerb (or median) to the opposite side of the road. For the driver, the pedestrian gap acceptance may affect the decision to yield or not.
- Position of a pedestrian, P: Whether the pedestrian is waiting from the kerb (0) or median (1), needs to be considered as an influencing variable. It is a binary discrete variable
- No. of waiting pedestrians, W: It’s a scale variable representing the number of people at the kerb or median waiting to cross at the point in time when there is an acceptable gap and before they begin to cross. A driver may be inspired to yield if the number of waiting pedestrians is high because it appears like they have waited too long. It is a discrete number.
- Direction of traffic flow, D_T_: This is a binary variable representing whether the road segment has traffic flow in one way or both directions. The direction of traffic flow (whether one (0) or both directions (1)) is more important to pedestrian gap acceptance than it may be for driver yielding. Unlike the pedestrian who has to simultaneously assess the viability of a gap considering vehicles moving in opposite directions, a driver only has to consider the actions of pedestrians at the crosswalk
- Presence of e-Police, E_P_: This is also a binary variable (Yes (1)/No (0)) representing the presence of e-Police or not. E-Police are the CCTV cameras used by the traffic police to monitor traffic flow and occurrence of any violations. Being aware of their presence affects yielding compliance for fear of the potential consequences.

The streets and the crosswalk spots that meet the characteristics of pedestrian and traffic flow volumes at mid-block uncontrolled crosswalks were selected for the study.

### Survey spots

Data for the study was collected from thirteen streets in Shanghai city, People’s Republic of China over a period of three months. Video data was analyzed to establish the number of vehicles that passed by that location per hour, the number of waiting pedestrians and their waiting time. The data collection took three months for a duration of 2 hours per day for each location. Table 1 gives a summary of the locations included in the study.

**Table 1:** Midblock crosswalk locations in Shanghai.

Number of traffic lanes	Direction	E-Police	Surveyed crosswalk	Nearest crossing street	Distance from the nearest crosswalk (m)
1	One way	No	Nancang Rd.	Sinan Rd.	100
2	One way	No	Yutian Rd.	Quyang Rd.	140
		Yes	Yongxin Rd.	Gonghexin Rd.	50
	Two way	No	Wulumuqi Rd.	Huashan Rd.	25
		Yes	Linshi Rd.	Nanhuayuan Rd.	50
3	One way	No	Shangcheng Rd.	Dongfang Rd.	160
	Two way	No	Linshi Rd.	Nanhuayuan Rd.	200
		Yes	Pingliang Rd.	Ninwu Rd.	85
4	One way	No	Changshou Rd.	Wanhangdu Rd.	180
		Yes	Changshou Rd.	Jiaozhou Rd.	160
	Two way	No	Zhenhua Rd.	Xincun Rd.	140
		Yes	Xincun Rd.	Zhenhua Rd.	180
5	Two way	Yes	Yinkou Rd.	Jiamusi Rd.	195

The data collected included the vehicles that yielded and those that didn’t. If a pedestrian crossed and stopped at the median of a wider street to wait for the next gap, the second crossing was considered a new crossing and recorded accordingly. Even those who arrived when the gap was acceptable and crossed were considered as waiting pedestrians but with 0s waiting time. A total of 11860 data points were recorded. Video data on vehicle yielding decisions were videotaped in real traffic conditions using a video camera mounted on unmanned aerial vehicle (UAV). Table 2 shows a sample of observations in the study. The authors confirm no specific permissions were required for the survey activities and the field studies did not involve endangered or protected species.

**Table 2:** Example of the surveyed data.

Sample No.	Yield (1) or not (0)	Time gap (s)	Number of lanes	Pedestrian number (person)	Position of pedestrian (0-kerbside, 1-median)	Traffic direction (0- 1way, 1- 2way)	E-Police monitoring (0- no, 1-yes)
1	1	4.64	2	1	0	0	1
2	1	2.36	1	1	0	0	0
3	1	2.14	2	3	0	0	1
4	1	6.44	1	1	0	0	0
5	1	5.34	2	5	1	1	0
6	1	13.35	3	2	0	0	0
7	1	13.28	2	2	1	0	0
8	0	1.67	1	1	0	0	0
9	0	3.53	8	7	1	1	0
10	1	10.56	2	3	0	0	0

## Model development and analysis

In a traffic situation, pedestrian actions will affect the driver’s next response and traffic flow in general regardless of the choices already made. If pedestrians chose to wait, the driver may proceed at current velocity and even accelerate if headway permits, or yield out of generosity considering the time pedestrian has waited. This study attempts to describe the observed driver yielding actions.

### Introduction to the logistic model

A statistical analysis was performed on the data to establish the probability of driver yielding to pedestrians and correlations between the variables. Multiple Linear Regression (MLR) was used to evaluate the effect of the influencing variables on the outcome variable and develop a statistical model to explain goodness of fit in the model. Logit model was used to describe the probability of a driver yielding considering the variables in the study. Sensitivity analysis was done to test the effects of the predictor variables on the outcome variable. The Bayesian Regression is an alternative binary regression approach to analyze this problem especially for problems with limited data or with prior knowledge to be used in the model. The Bayesian linear regression method can introduce prior information and show uncertainty at the same time [42–44]. However, in this study, we had no prior knowledge to be used and the data in this study is large. Therefore, maximum likelihood evaluation was suitable for our study as it has no restrictions on the independent variables [45].

The vehicle yielding variable in this study is a binary outcome variable where responses take the values 1 and 0 (yield or not yield respectively). The expected outcome value is the probability that the variable takes the value 1, i.e., the probability of yielding depending on the independent variables which, in this study, are both categorical and scale. The proposed model is a discrete choice model where the driver has to decide whether to yield and allow the waiting pedestrians to cross or not. The, logarithmic transformation of the binary outcome is employed to transform the data to categorical to be able to express a non-linear relationship in a linear way. Logistic regression employs this principle to express the multiple linear regression equation in logarithmic terms (called the logit) and thus overcomes the problem of violating the assumption of linearity. In this study, the logistic regression model is the most suitable for modelling this outcome.

The logistic regression model is considered most suitable for modelling the phenomena because of the binary outcome variable. The model is derived from the multiple linear regression function, *Y*, written as the linear sum of β_0_ plus the products of coefficients, βn and the corresponding *Xn* variables, the independent variables of interest expressed as Eq (1).

$$Y=\alpha +\beta_{1}X1+\beta_{2}X2+\cdots \;\beta_{n}Xn$$ (1)

where: *n* is the number of independent variables, α: constant (y intercept); *β* is beta coefficients and *Xn* is the *n*^*th*^ predictor (independent) variable. The variable *Y* is obtained from a multiple linear regression model. The effect of selected variables on the vehicle yielding behaviour is described with the help of multiple linear regression model. The probability of yielding and not yielding denoted *p(y)* and *p (1-y)* respectively, then the probability of yielding can be calculated using the Logit function, as shown in Eq (2).

$$p(Y)=\frac{1}{1+e^{-Y}}$$ (2)

### Tests of the application presuppositions of the logistic model

#### Multi-collinearity test

Perfect collinearity between predictor variables makes it impossible to obtain unique estimates of the regression coefficients due to an infinite number of possible combinations of coefficients that would work equally well. Multi-collinearity between predictor variables poses a challenge in assessing the individual importance of a predictor and its effect on the outcome. The Variance Inflation Indicator (VIF) and tolerance factor are indicators that show whether a predictor has a strong linear relationship with the other predictor(s). Generally, if the largest VIF is greater than 10 or if the average VIF is substantially greater than 1 then the regression is most likely to be biased. Further, when Tolerance is below 0.1 then multi-collinearity is likely [46].

From the Table 3, the variance inflation factor (VIF) for all the independent variables was between 1.053 and 1.388, while tolerance was between 0.720 and 0.992. If the Tolerance is less than 0.1 or the VIF is greater than 10, then there is multi-collinearity. In this case, the tolerance is much greater than 0.1, and the variance expansion factor is less than 10, so there is no multi-collinearity. Therefore, the data is suitable for MLR analysis.

**Table 3:** Multiple linear regression.

Model	Unstandardized Coefficients		Standardized Coefficients	t	Sig.	Collinearity Statistics	
	B	Std. Error	Beta			Tolerance	VIF
(Constant)	.716	.008		84.981	.000		
time_gap	.048	.001	.524	78.430	.000	.944	1.059
number_of_lanes	-.186	.003	-.536	-70.152	.000	.720	1.388
pedestrian_number	.022	.003	.056	8.596	.000	.992	1.008
peds_position	.003	.007	.003	.380	.704	.950	1.053
traffic_dir	-.024	.007	-.027	-3.519	.000	.738	1.355
ePolice	.233	.006	.250	37.358	.000	.940	1.064

#### Linearity test

This was done using the Box-Tidwell transformation. The method verifies whether there is a linear relationship between the continuous independent variables and the logit conversion value of the dependent variable. If the interaction is statistically significant (i.e. p < 0.05), there is no linear relationship between the corresponding continuous independent variable and the dependent variable logit conversion value. The results are presented in the Table 3.

In the Table 4, all the transformed continuous independent variables in the model (LN_time_gap, LN_number_of_lanes, and LN_pedestrians_number) have a p-value >0.05, indicating that they each had a linear relationship with the outcome variable. Thus, having established that the independent variables have no multi-collinearity and have individual linear relationship with the outcome variable, a logit model can be developed and used to predict vehicle yielding accordingly.

**Table 4:** Box-Tidwell test for linearity.

	B	S.E.	Wald	df	Sig.	Exp(B)
time_gap	2.600	.292	79.335	1	.000	13.459
number_of_lanes	-8.108	.643	159.006	1	.000	.000
pedestrian_number	.903	.385	5.506	1	.019	2.468
peds_position(1)	3.043	.196	239.952	1	.000	20.965
traffic_dir(1)	.296	.166	3.189	1	.074	1.345
ePolice(1)	12.116	.451	722.112	1	.000	182797.584
LN_time_gap by time_gap	.144	.112	1.655	1	**.198**	1.155
LN_no_lanes by number_of_lanes	-.295	.336	.773	1	**.379**	.745
LN_peds_no by pedestrian_number	-.086	.205	.177	1	**.674**	.917
Constant	1.195	.614	3.790	1	.052	3.303

### Binary logistic model

The proposed model is a discrete choice model where the drivers have to make one of two choices, whether to yield or not considering the predictor variables identified. For developing the logit model, 75% of the data was used to establish the contribution of each of the predictor variables to the vehicle yielding decision. The remaining 25% of the data collected was used to validate the model.

The descriptive statistics of this model, presented in Table 5 below, shows that all the predictor variables (gap size, number of lanes, number of waiting pedestrians, pedestrian position and ePolice) were found to be statistically significant with a p-value <0.05 but, the traffic flow direction which had a p-value >0.05 failed the test of statistical significance. This implies that there is no significant correlation between traffic flow direction vehicle yielding and any change in the variable will not affect the outcome variable. Therefore, the traffic flow direction variable is omitted, and the logit model is re-calibrate, as shown in Table 6.

**Table 5:** Logit model.

	B	S. E.	Wald	df	Sig	Exp(B)
Constant	1.311	.170	59.802	1	.000	3.710
time_gap	2.957	.101	855.600	1	.000	19.241
number_of_lanes	-8.576	.292	860.791	1	.000	.000
pedestrian_number	.742	.062	145.278	1	.000	2.101
peds_position(1)	3.008	.190	250.261	1	.000	20.244
traffic_dir(1)	.302	.159	3.616	1	.057	1.353
ePolice(1)	11.971	.428	780.484	1	.000	1.58E05

**Table 6:** Revised logit model.

	B	S. E.	Wald	df	Sig	Exp(B)
Constant	1.291	.193	44.758	1	.000	3.636
time_gap	2.937	.117	635.052	1	.000	18.862
number_of_lanes	-8.462	.332	650.584	1	.000	.000
pedestrian_number	.730	.071	105.590	1	.000	2.075
peds_position(1)	3.008	.224	180.393	1	.000	20.256
ePolice(1)	11.913	.494	581.604	1	.000	1.49E05

Further, the Odds Ratio represented by Exp(B) in Table 6, is a statistic that represents the change in odds of a predictor variable influencing the change in the outcome variable and its reference pivot point is 1. When an odds ratio value is greater than 1, then it indicates that as the predictor increases, the odds of the outcome occurring increase. Conversely, a value less than 1 indicates that as the predictor increases, the odds of the outcome occurring decrease [46]. Given that the value of 1 is the threshold at which the direction of the effect changes, it follows that odds ratios of 1 (or near 1) indicates the least effect (or none) on the outcome variable by the particular predictor. In this study, we can say that the odds of a motor vehicle driver yielding were 18.862 times higher with each increase in the gap size while the odds of yielding decrease with the increase in the number of lanes. However, there will be minimal effect from the number of waiting pedestrians (2.075) because the odds ratio is very close to 1.

The test results in Table 6 also shows the Beta coefficients (B), standard errors (SE) and ‘p’ values for the model logit. Based on the results presented above, and omitting the variables that were found to be statistically insignificant, the regression model can be written as:

$$Y=1.291+(2.937*G_{s})-(8.462*L)+(.730*W)+(3.008*P)+(11.913*e_{P})$$ (3)

**Fig 1:**
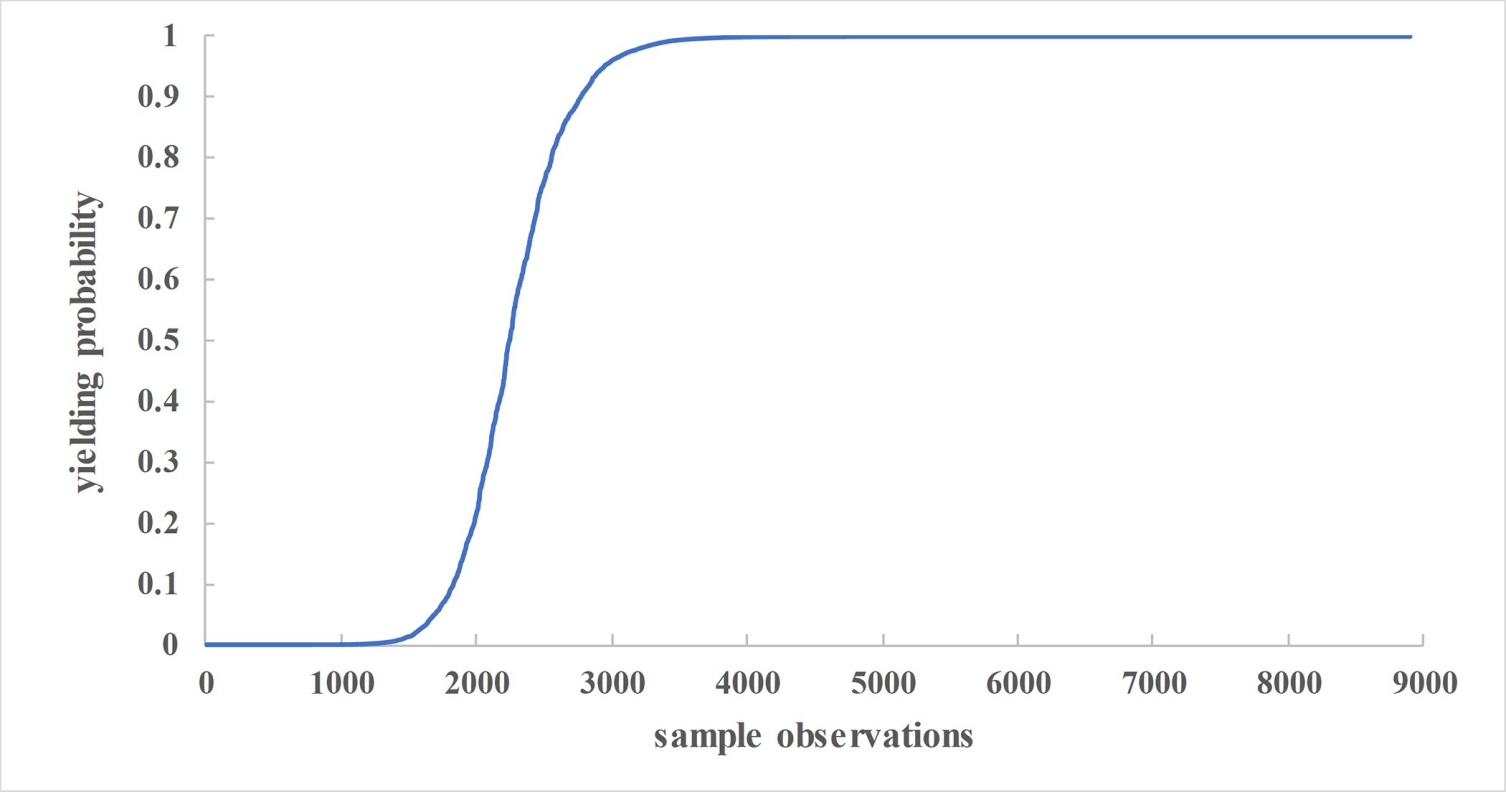
Fitted vehicle yielding logit model.

Thus the probability of drivers yielding to pedestrians in this study can be expressed through the following probability function. Fig 1 presents the results of the fitted model based on the field data.

$$p(Y)=\frac{1}{1+e^{-Y}}$$ (4)

### Validity of the model

The 25% of field data was used to validate the logit model. The validity of this model was undertaken to establish its reliability by comparing the observed instances of yielding and the predicted probability of yielding based on the logit model. Probability of 0.5 and above denotes success (Yield) and less than 0.5 denotes No yield. Overall, the logit model was able to accurately predict a total of 2844 (95.92%) out of 2965 sample observations of which 2134 (97.58%) vehicle yields and 710 (91.26%) non-yields. Table 7 is a summary.

**Table 7:** Model validation.

	Observed	Predicted	Predicted accurately	Percentage accurate
Yield	2187	2202	2134	97.58
Not yield	778	763	710	91.26
Total	2965	2965	2844	95.92

Further, a Paired t-test results in Table 8 shows that there is no significant difference between results estimated by the proposed model and that from simulation (p-value = 0.173 > 0.05), indicating that the accuracy of the proposed vehicle yielding model is acceptable. Therefore, with an accuracy of 95.9%, this model can be used to predict the circumstances under which a driver can yield to pedestrians given the influencing factors considered in this study.

**Table 8:** Paired sample t-test.

Paired Differences					t	df	Sig. (2-tailed)
Mean	S. D.	Std. Error Mean	95% C.I. of the Difference				
			Lower	Upper			
-.0051	.2020	.0037	-.0123	.0022	-1.364	2964	.173

## Discussion

Sensitivity Analysis is used in statistical modeling to analyze how different values of a set of predictor variables affect an outcome variable under specific varying conditions. In this study Sensitivity analysis was done on the predictor variables that were statistically significant, i.e.: gap size, number of lanes, number of waiting pedestrians, position of pedestrians and ePolice. From the five variables that were statistically significant, two binary categorical variables (pedestrian position and ePolice) were weakly correlated with the outcome variable while gap size, number of traffic lanes and number of waiting pedestrians were strongly correlated with the outcome variable. This analysis examines how the variables affect the outcome variable. The basic input parameters are: gap size (8s), number of traffic lanes (5), and the number of waiting pedestrians (3). The sensitivity of each of the continuous variable was determined with respect to each of the alternative options of the binary variables, pedestrian position (0, kerb vs. 1, median) and presence of ePolice (1, present, vs. 0, absent). A 0.5 cut-off is used to determine the threshold for the probability of success. Thus, the driver is predicted to yield if probability is ≥ 0.5 and failure to yield is determined by a probability of <0.5.

### Gap size

**Fig 2:**
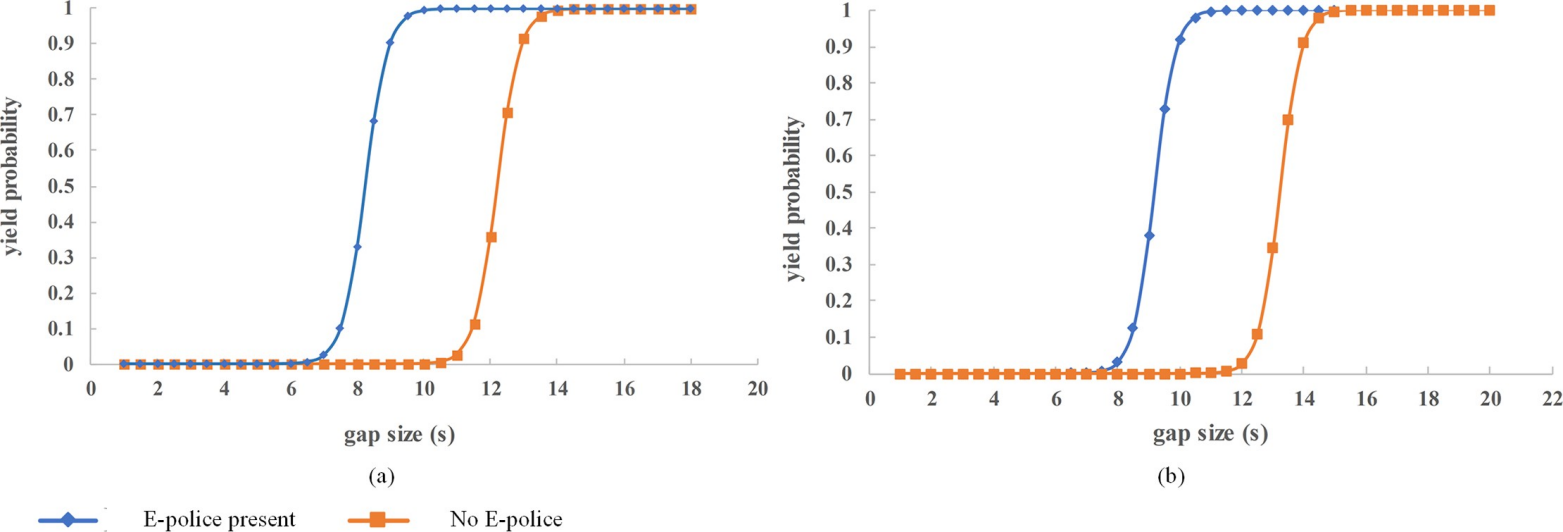
Influence of gap size on vehicle yielding.

In this study the gap size represents the time an oncoming vehicle will traverse from the point of observation to the spot where the crosswalk is located. It is an important influencing factor as it determines whether pedestrians will attempt to cross or reject the available gap. In this analysis, we considered the influence of different gap sizes, between 0 and 30 seconds, on the outcome variable of the model. The results were plotted in Fig 2.

Fig 2 shows the influence of the gap sizes on the probability of vehicle yielding for a 5-lane road considering the position of waiting pedestrians and presence of ePolice. For a pedestrian waiting at the kerbside (Fig 2A), the model predicts that drivers are likely to yield when the gap is at least 9.5s if ePolice is present and a bigger gap of 13.5s if ePolice is absent. When pedestrians wait at the median (Fig 2B), vehicles yield at 8.5s gap with ePolice present and 12.5s gap without ePolice. Generally, the model predicts that there is a higher probability of vehicles yielding if pedestrians wait at the median and if ePolice was present.

**Fig 3:**
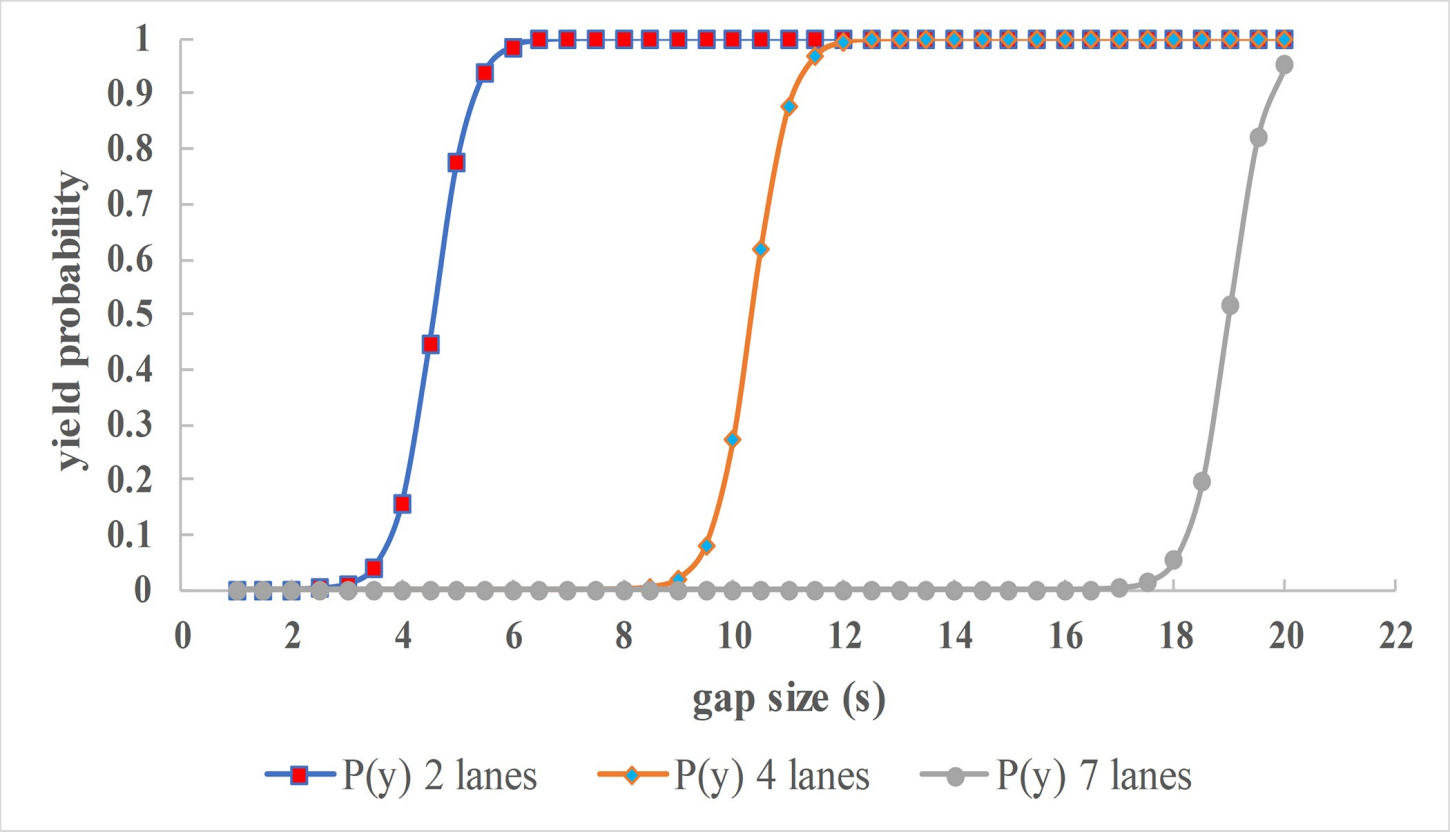
Effect of gap size considering number of lanes.

For a slow traffic flow on a single-lane road, a small gap can allow pedestrians to cross but the same gap on a 5-lane road is risky for pedestrian safety. Therefore, the number of lanes also has a profound effect on the effect of gap size on probability of yielding as shown in Fig 3.

Fig 3 considers a scenario where there is no ePolice and pedestrians wait at the kerbside of 2, 4 and 7-lane roads respectively. It is clear that vehicles are more likely to yield with a lower gap size if the number of lanes is smaller than if the lanes were many. For 2-lane roads, vehicles yield at 5s gaps, for 4-lanes 10.5s and 19s gaps for 7-lane roads respectively. This can be explained by the fact that roads with fewer lanes tend to be in heavily populated urban areas that have slow moving less-risky vehicles while roads with 4 or more lanes tends to have higher traffic flow speeds. Larger gaps are likely to be acceptable to the pedestrians who may be already crossing when a vehicle approaches, hence the higher yielding probability.

Given that the gap size is a function of distance and the speed, it is affected by the approach speed of the oncoming vehicle [20]. The results above are consistent with [34] where 75% of vehicles yielded on a 2-lane road where vehicle speed was 20mph while a 4-lane street had only 9% yielding. There is therefore a need to have traffic calming interventions that preserve the expected efficient traffic flow and ensure pedestrian safety on multi-lane roads without solely relying on the drivers’ judgement and goodwill. Signal control can enforce yielding for roads with more than 4 lanes. In the absence of signal control, Median Island can be introduced if space allows.

### Number of lanes

**Fig 4:**
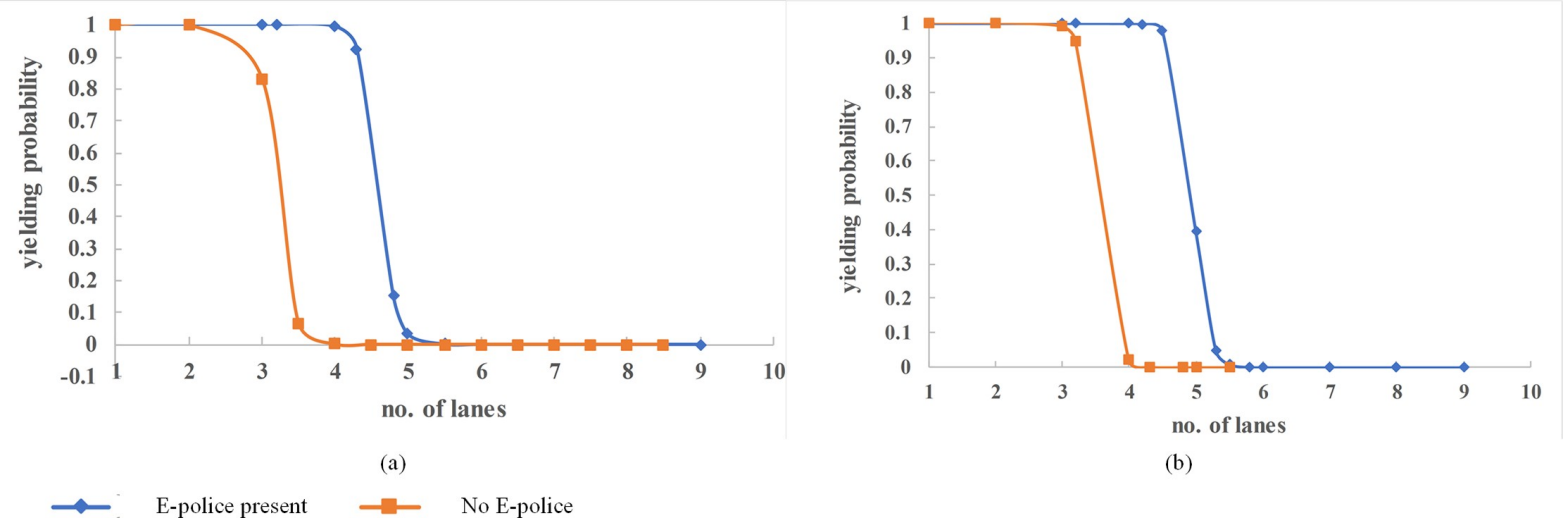
Effect of number of lanes on vehicle yielding.

The number of lanes in this study represents the number of lanes going in one direction of traffic flow. The higher the number of lanes, the longer the distance to traverse, and the harder it is for pedestrians to assess the parallel gaps from multiple oncoming vehicles as well as the higher risk of tragic conflicts. Besides, the presence of ePolice and the position of pedestrians (whether at kerbside or median island), it also has an effect on whether drivers yield or not. Generally, as Fig 4 shows, the probability of yielding is higher with fewer lanes and diminishes as the number of lanes increases but the presence of ePolice and position of pedestrians has some influence. This implies that drivers on wider multi-lane roads are reluctant to yield to pedestrians.

From Fig 4(A), the probability of yielding to pedestrians waiting at kerbside dips sharply from a high of 92% for 4.3-lane roads to a low of less than 1% for 5-lane roads when ePolice is present whilst without ePolice, the probability of yielding dips from a high of 83% for 3-lane roads to a low of less than 1% for 3.5-lane roads. Above the 50% probability cutoff point, drivers are predicted to yield on 3-lane roads (83%) without ePolice presence and 4.3-lane roads (92%) with ePolice respectively. Clearly, the presence of ePolice is an incentive to yield even if the number of lanes are higher.

In Fig 4(B), for pedestrians waiting at the median island the probability of yielding dips sharply from a high of 98% for 4.5-lane roads to a low of 7% for 5.3-lane roads when ePolice is present whilst without ePolice, the probability of yielding dips from a high of 95% for 3.2-lane roads to a low of less than 1% for 4-lane roads. At 50% probability cutoff point, drivers are predicted to yield on 4-lane roads (73%) with ePolice presence and 3-lane roads (64%) without ePolice respectively. Therefore we can conclude that drivers are more likely to yield when pedestrians are at median and where ePolice is present than at kerbside without ePolice.

This scenario described by the model conforms to pedestrian gap acceptance scenario where pedestrians readily accept gaps on road segments with fewer lanes and shorter crossing distances [34]. The higher the number of lanes, the longer the distance a pedestrian needs to traverse and the more complicated it is in assessing viability of available gaps against multiple vehicles on multiple lanes. Therefore, though it is a designated crosswalk, pedestrians would be reluctant to cross. Considering the varied characteristics and personalities of drivers, it may not be wise to leave yielding to the goodwill and judgement of drivers for wider roads. We therefore recommend a median island for roads with more than 4 lanes and if there is no space for it, signal control in addition to ePolice can be introduced as a safety intervention [47, 48].

### Number of waiting pedestrians

**Fig 5:**
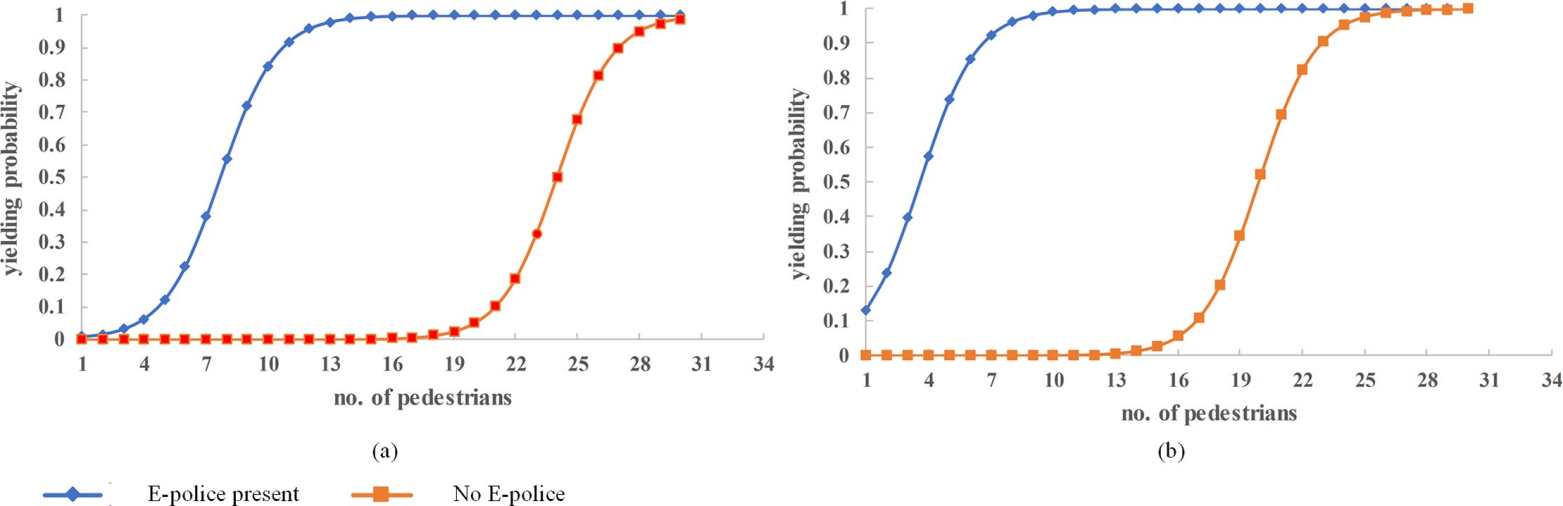
Effect of number of waiting pedestrians.

A crowd at the kerb or street median at unsignalized crosswalk can be an indicator that they have waited for a safe gap for some time and prompt a less aggressive driver to yield. Such pedestrians can become impatient and develop a higher propensity for risky crossing behavior. An analysis of the influence of the number of waiting pedestrians on the model outcome variable was done and the results are shown in Fig 5.

Fig 5 shows that the probability of a driver yielding is predicted to be higher when the number of pedestrians is higher for pedestrians waiting at the median island with ePolice present compared to those waiting at the kerbside. In Fig 5(A) with pedestrians waiting at the kerbside and considering the 0.5 probability cutoff point, vehicles yield when there are more than 8 pedestrians with ePolice present but will only yield when more than 24 pedestrians are waiting if ePolice is absent. With pedestrians waiting at the median island and ePolice present, Fig 5(B), vehicles will yield when at least 5 pedestrians are present but only yield when there is at least 20 pedestrians if no ePolice present.

From the foregoing, it is predicted that drivers are sensitive to large numbers of waiting pedestrians, especially at the median, and the presence of ePolice compared to smaller numbers and the absence of ePolice. According to the Situational Action Theory [49], people who have a tendency to commit crimes may obey the law for fear of potential legal consequences. The presence of ePolice is an incentive for drivers to do the right thing under the circumstances and yield [37]. Thus we can recommend that where space allows, wider roads to have median islands and installation of ePolice to enhance the safety of pedestrians using the crosswalk.

## Conclusion

The aim of this research was to investigate and model the vehicle yielding behavior at unsignalized midblock crosswalks. We focused on environmental factors that can be controlled by urban roads transportation administrators. The following Six variables were identified as having influence on the vehicle yielding decisions: gap size (seconds), number of lanes, pedestrian number (persons), position of pedestrian on the street, traffic flow direction, and E-Police. 11,860 samples of observation data were collected from 13 streets ranging from single lane one-way to 5 lane bi-direction roadway. Multiple Linear Regression and Logit models were used to analyze the data. Traffic flow direction, which had a p-value of >0.05, was found not to be statistically significant. The model developed had a 95.9% accuracy in predicting the vehicle yielding behavior. From the sensitivity analysis that was conducted the following observations can be made:

1. Vehicles were more likely to yield on roads with fewer lanes (less than 5 lanes) than roads with more than 4 lanes.
2. Vehicles were more likely to yield with larger gaps than smaller gaps.
3. There was a direct relationship between gap size and number of lanes where vehicles yielded with smaller gaps on single lanes compared to multiple lanes. An 8-lane road required at least 18s gap size to yield.
4. Vehicles were likely to yield if there were many waiting pedestrians than if they we fewer. The probability was even higher if they waited at the median than at the kerb.
5. Drivers appeared to be sensitive to the presence of ePolice and yielded more than when there was no ePolice. This could be an indicator that they were aware of the pedestrians’ right of way but needed an incentive to comply.

This study was limited to observing drivers’ actions (whether they yielded or not) based on observed environmental factors (gap size, number of lanes, number of pedestrians and their positions, traffic flow direction and presence of ePolice). Naturally, factors related to their character and personality (which relies largely on inaccurate self-reporting), was a contributor but it wasn’t possible to verify that. A future study could include modelling the interactions of both pedestrians and drivers behavior at the midblock crosswalks considering competition between the two in using the road segment.

## Supporting information

S1 Table Original data. (XLSX)
